# 3D-ASC-Exos as a novel drug carrier for glioma treatment

**DOI:** 10.3389/fcell.2025.1677555

**Published:** 2025-09-25

**Authors:** Xintong Han, Xin Li, Xinrui Yan, Yu Zhang, Chang Liu, Wei Liu, Rui Han, Yaxin Li, Jianming Li, Naichao Diao, Boyin Jia, Rui Du

**Affiliations:** ^1^ College of Agriculture, Yanbian University, Yanji, China; ^2^ College of Animal Science and Technology, Jilin Agricultural University, Changchun, China; ^3^ Jilin Province Sika Deer Efficient Breeding and Product Development Technology Engineering Research Center, Jilin Agricultural University, Changchun, China

**Keywords:** antler, 3D-ASC-Exos, glioma, drug carrier, TMZ

## Abstract

**Introduction:**

It is known that extracts from deer antler stem cells have an inhibitory effect on glioma growth. Therefore, it is hypothesized that exosomes derived from deer antler stem cells (ASC-Exos) can be used as a new drug carrier for the treatment of glioma.

**Methods:**

To begin with, we established a 3D culture system to obtain more exosomes and characterized the 3D-ASC-Exos. Subsequently, we loaded TMZ into 3D-ASC-Exos. Evaluate the effects of 3D-ASC-Exos loaded with TMZ on glioma cell proliferation, migration, invasion, and apoptosis at the cellular level. Additionally, the safety and efficacy of 3D-ASC-Exos loaded with TMZ against glioma were evaluated using tumor-bearing mice.

**Results:**

Compared with the 2D-ASC-Exos obtained from the traditional 2D culture, the 3D-ASC-Exos obtained from our constructed 3D culture system were concentrated nearly 30 times in the culture medium volume, which was more convenient for subsequent puriffcation. The two forms of ASC-Exos had similar morphologies and surface markers, but 3D-ASC-Exos were enriched with more miRNAs related to tumor suppression. In vitro experiments demonstrated that 3D-ASC-exosomes loaded with temozolomide (TMZ) inhibited the proliferation, migration and invasion abilities of glioma cells and promoted the apoptosis of glioma cells. The vivo tumor-bearing mouse model demonstrated that 3D-ASC-Exos loaded with TMZ exerted tumor-suppressive effects by inhibiting tumor growth and promoting tumor apoptosis. Meanwhile, the treatment with 3D-ASC-Exos loaded with TMZ caused no damage to the various tissues and organs of mice compared with the TMZ group.

**Discussion:**

3D-ASC-Exos can be used as a novel drug carrier for glioma treatment. The development of 3D-ASC-Exos as a drug carrier not only provides a better strategy for tumor treatment, but also demonstrates the broad potential of exosomes in targeted tumor therapy.

## 1 Introduction

Exosomes are nano-sized vesicular structures secreted by cells, and they contain bioactive molecules such as proteins, RNA, and DNA inside (van Niel et al., 2022). An increasing number of studies show that exosomes derived from different mesenchymal stem cells (MSCs), such as bone marrow mesenchymal stem cells (BMSCs), adipose mesenchymal stem cells (AMSCs) and human umbilical cord mesenchymal stem cells (hUCMSCs) play significant roles in inhibiting the progression of breast cancer, colon cancer and bladder cancer, respectively (Safari et al., 2023; Zhang et al., 2020; Wu et al., 2013). In addition, the anticancer ability of MSCs-derived exosomes as drug carriers has been confirmed in many studies. Compared with simple drug treatment, the therapeutic effect of exosome-loaded drugs was better, and it reduced the adverse effects on the body caused by the intake of pure drugs (Hu et al., 2022). Therefore, MSCs-derived exosomes have emerged as an ideal carrier for drug delivery. However, the latest research has found that MSCs have pro-cancer effects such as promoting tumor angiogenesis, reshaping the immunosuppressive microenvironment, and transmitting oncogenic miRNAs through exosomes (Guo et al., 2020; Liang et al., 2021). Therefore, it is necessary to seek safer stem cell resources.

Antler is the only mammalian organ that can regenerate periodically (Nieto-Diaz et al., 2012). Antler regeneration is a process based on antler stem cells (ASCs). Antler regeneration consumes approximately 3.3 million ASCs each year. In less than 60 days, antler weighing 10 kg is formed (Li et al., 2023). The growth rate of antler is 30 times that of tumors. But antler and its surrounding tissues do not undergo cancerous transformation (Jia et al., 2021). Therefore, ASCs have a strong resistance to tumors. ASCs preferentially migrate towards tumors and regulate the progression of tumor cells through paracrine rather than cellular means. Among them, ASC-Exos play a major role in paracrine secretion (Liu et al., 2023). Glioma is a common type of malignant brain tumor (Fernandes et al., 2017). The current common treatment approach is surgical resection, supplemented by temozolomide (TMZ) for radiotherapy and chemotherapy (Jezierzański et al., 2024). However, due to primary or acquired drug resistance mechanisms, the efficacy of TMZ is limited. Compared with TMZ, the extract from the tip of antler exerts anti-glioma effects by cell cycle arrest, and has no toxicity to non-cancer cells (Chonco et al., 2021). Therefore, we speculate that ASC-Exos can be used as nano-drugs or drug delivery carriers for the treatment of glioma.

At present, the relatively mature MSCs culture process in clinical practice is the 2D culture mode. This mode, due to high production costs and cumbersome operations, cannot meet the demands of large-scale production. In recent years, an increasing number of research reports have indicated that 3D culture mode is more similar to the microenvironment within the body. The 3D culture mode can obtain higher-quality exosomes by controlling the parameters (such as temperature, pH, etc.) during the cell growth process, while maintaining the surface characteristics of the exosomes (Phan et al., 2018). In addition, the exosome population obtained in the 3D culture system is more diverse and freer from serum protein contamination (Storm et al., 2016; Gobin et al., 2021; Yan et al., 2018). However, reports on the optimization of 3D culture systems for ASCs are still quite limited. In this study, we employed a 3D culture system to produce high-quality and high-concentration 3D-ASC-Exos. And investigate the effects of 3D-ASC-Exos loaded with drugs (TMZ) *in vitro* on the proliferation, migration, invasion and apoptosis of glioma cells. Subsequently, we further evaluated the therapeutic effect and safety of 3D-ASC-Exos loaded with drugs (temozolomide) on tumor-bearing mice. In conclusion, 3D-ASC-Exos efficiently prepared through the 3D culture system can be used as drug delivery carriers to treat glioma.

## 2 Materials and methods

### 2.1 Isolation and identification of ASCs

Collagenase I (64 mg), collagenase II (52 mg), and collagenase IV (44 mg) were dissolved together in 80 mL of DMEM and then filtered through a 0.22-micron membrane to prepare the tissue digestion enzyme for later use. We isolated the translucent mesenchymal layer from the tip of the antler. After digestion in a tissue digestion mixed enzyme, it was used for primary cell culture. We identified the cell morphology, trilineage differentiation ability and surface markers of the third-generation ASCs.

### 2.2 Cultivation of ASCs in 3D culture systems

Fetal bovine serum was centrifuged at 4 °C, 160,000×g, and 16 h. Collect the clear liquid on the top layer after centrifuging the fetal bovine serum, and this liquid is the low exosome serum. The inner diameter of the fibers in the medium-sized hollow fiber bioreactor was approximately 200 μm. The fibrous material was composed of polysulfone (PS) and polyvinylidene fluoride (PVDF). Each culture tube could hold approximately 20 mL of culture medium. The 3D culture system was successively circulated internally and externally with enzyme-free and sterile PBS, DMEM, and complete medium containing 10% fetal bovine serum for 24–48 h. Then, the ASCs were inoculated into the outer circulation culture cylinder of the 3D system and repeatedly tapped until the cells adhered evenly. We would invert the culture tube up and down every 30 min, and then connect it to the complete culture medium after 2 h and 30 min. The sugar consumption in the complete culture medium should be checked every other day to replace it with fresh complete culture medium in a timely manner.

### 2.3 Isolation and identification of 3D-ASC-Exos

Firstly, ELISA and PCR were respectively used to detect the contents of *Chlamydia* and *Mycoplasma* in the supernatants of 2D-ASCs and 3D-ASCs. Then, the supernatant was subjected to gradient centrifugation to harvest exosomes. Briefly, the process involved centrifugation at 4 °C for 15 min at 300×g, followed by 30 min at 2000×g, then 30 min at 10,000×g, and finally 2 h at 120,000×g. Finally, 2D-ASC-Exos and 3D-ASC-Exos were identified by transmission electron microscopy, nanoparticle tracking, and WB.

### 2.4 Small RNA sequencing of 3D-ASC-Exos

Firstly, we used the exoRNeasy Maxi kit to purify the RNA of 2D-ASC-Exos and 3D-ASC-Exos. Secondly, the qualified 2D-ASC-Exos and 3D-ASC-Exos were subjected to library construction using the Small RNA Sample Pre Kit. Finally, the obtained data were subjected to sRNA length screening, reference sequence alignment analysis, known miRNAs analysis, repeat sequence alignment, miRNAs differential expression analysis, and GO/KEGG enrichment analysis of candidate target genes. The raw transcriptome data from this study have been submitted to the NCBI Gene Expression Omnibus with accession number GSE305022 (https://www.ncbi.nlm.nih.gov/geo/query/acc.cgi?acc=GSE305022).

### 2.5 *In vitro* evaluation of the effect of 3D-ASC-Exos loaded with TMZ on inhibiting glioma cells

After labeling TMZ with fluorescein isothiocyanate (FITC), 3D-ASC-Exos (100 μg/mL) were co-incubated with TMZ (10 μg/mL) at room temperature for 18 h. The 3D-ASC-Exos loaded with TMZ were separated from the supernatant by ultracentrifugation. The fluorescence intensity in the supernatant before and after centrifugation was detected to determine the loading amount. The 3D-ASC-Exos loaded with TMZ were placed in dialysis bags, and the percentage of drug release in the solution outside the dialysis bags was detected at 0 h, 4 h, 8 h, 12 h, 16 h, 20 h and 24 h respectively. 3D-ASC-Exos (100 μg/mL) and the positive drug TMZ (10 μg/mL) were used as controls. CCK8 was used to evaluate the proliferation ability of U87 and A172. At the same magnification as the original image (200X), the quantitative method for scratches was to divide the width of the scratch by the total width of the image. Cell scratch and Transwell were used to evaluate the migration levels of U87 and A172. Transwell was used to evaluate the invasive ability of U87 and A172(In addition to the materials used in the migration assay, Matrigel was also used). TUNEL was used to evaluate the apoptosis levels of U87 and A172. Q-PCR and WB were used to detect the expression of BAX, BCL2, P53 and EGFR in U87 and A172.

### 2.6 *In vivo* evaluation of the safety and efficacy of 3D-ASC-Exos loaded with TMZ in anti-glioma treatment

All experimental procedures involving animals were conducted in accordance with the ethical policies and guidelines approved by the Animal Ethics Committee of Jilin Agricultural University (Approval no. 20230315010). Due to technical limitations, *in situ* tumor modeling was not used to treat tumor-bearing mice in this experiment. Established a U87 tumor-bearing mouse model. Twelve female BALB/c nude mice were randomly selected. Dilute 5 × 10^6^ U87 cells in 0.15 mL PBS. Then, inoculate them subcutaneously 0.5 cm lateral to the right thigh. One week later, the tumor-bearing nude mice were treated. The samples were divided into the following four groups: 3D-ASC-Exos group (100 μg/mL), TMZ group (10 μg/mL), 3D-ASC-Exos + TMZ group (100 μg/mL), and Control group (PBS). Treat once every other day for 20 consecutive days. During the treatment period, the body weight and tumor size of the nude mice were measured every other day, and the measurement curves were plotted. At the end of the experiment, the mice were anesthetized with chloral hydrate. The tumor was removed, photographed and weighed. Routine blood tests were used to evaluate physiological indicators. Blood biochemistry was used to assess liver and kidney functions. HE staining was used to evaluate the injury levels of the heart, liver, spleen, lung and kidney. HE staining was used to evaluate the cellular morphology and tissue structure of tumors. TUNEL staining was used to detect the level of apoptosis in tumors. Ki67 was used to detect the level of cell proliferation. Immunohistochemical detection was performed to examine the expression of BAX, BCL2, P53 and EGFR.

### 2.7 Analysis of data

We used the software SPSS to conduct statistical difference analysis on all the data. The analysis method of this experiment was one-way analysis of variance. GraphPad and Origin were used to plot the data. Statistical differences were indicated by P. Within a group, P < 0.05 was marked with “*”, P < 0.01 with “**”, and P < 0.001 with “***”.

## 3 Results and discussion

### 3.1 Identification of ASCs

**FIGURE 1:**
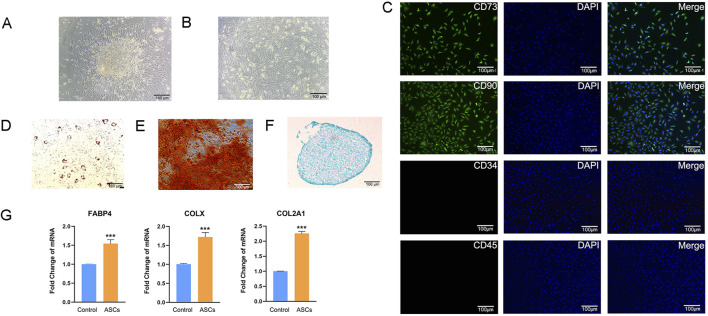
Isolation and identification of ASCs. **(A)** ASCs emigrated from tissues. **(B)** ASCs cultured to the third generation. **(C)** Immunofluorescence analysis of surface markers of ASCs. **(D)** Oil Red O staining of ASCs induced to adipogenic differentiation after 14 days. **(E)** Alizarin Red staining of ASCs induced to osteogenic differentiation after 21 days. **(F)** Alcian Blue staining of ASCs induced to chondrogenic differentiation after 14 days. **(G)** Q-PCR analysis of the expression levels of three marker genes after induction of differentiation (n = 3). Data were presented as mean ± SD. One-way ANOVA combined with Tukey’s HSD test and Bonferroni correction was used. *P < 0.05, **P < 0.01, ***P < 0.001.

We successfully obtained primary ASCs by collagenase digestion (Figure 1A). The proliferation ability of ASCs cultured up to the third generation remained good, and their morphology was mostly long spindle-shaped (Figure 1B). The IFA results of ASCs showed that CD73 and CD90 were highly expressed, while CD45 and CD34 were not expressed (Figure 1C). This result ruled out the possibility of other types of cells being mixed in the obtained cells, further clarifying the purity and specificity of the isolated cells. The trilineage differentiation staining results of ASCs showed that ASCs successfully differentiated into adipocytes, osteocytes and chondrocytes, respectively (Figures 1D–F). Q-PCR results indicated that the three types of cells after induction differentiation expressed the marker genes of adipocytes, osteocytes and chondrocytes, respectively (Figure 1G). The above results indicate that the ASCs we isolated and extracted meet the identification criteria for MSCs set by the International Society for Stem Cell Research (Dominici et al., 2006). The multipotent differentiation potential of ASCs was closely related to their paracrine function, and as an important carrier of paracrine action, the functional activity of ASC-Exos may be associated with the biological characteristics of ASCs. We identified that the extracted ASCs were MSCs, and this result provided a solid cell biological foundation for the subsequent exploration of 3D-ASC-Exos as drug carriers in the treatment of glioma.

### 3.2 3D culture of ASCs and identification of 3D-ASC-Exos

**FIGURE 2:**
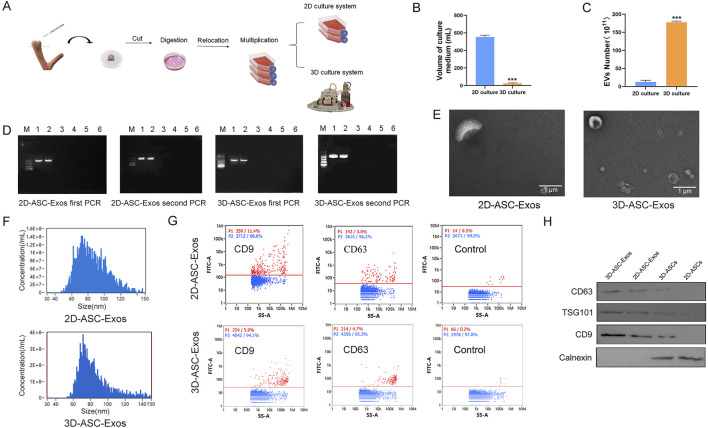
Isolation and identification of 3D-ASC-Exos. **(A)** Technical routes for 2D and 3D culture of ASCs. **(B)** Volume of culture medium used for the production of 4 × 10^11^ ASC-Exos (n = 3). **(C)** Number of exosomes produced per 10^8^ ASCs. **(D)** ELISA detection of *Chlamydia* content in the supernatants of 2D-ASCs and 3D-ASCs. **(E)** Transmission electron microscopy images of the two types of ASC-Exos. **(F)** Nanoparticle tracking analysis of the diameter size distribution of the two types of ASC-Exos. **(G)** Nanoflow cytometry analysis of the expression of markers CD63 and CD9 on the two types of ASC-Exos. **(H)** WB analysis of the expression of markers CD63, TSG101, CD9 and Calnexin on the two types of ASC-Exos. Data were presented as mean ± SD. One-way ANOVA combined with Tukey’s HSD test and Bonferroni correction was used. *P < 0.05, **P < 0.01, ***P < 0.001.

Hollow fiber bioreactors are a type of bioreactor used for 3D cell culture and can also be employed to obtain high concentrations of exosomes. The hollow fiber cell culture bioreactor mimics the human physiological circulatory system, providing a growth environment for cells that is closer to that of a living body (Cao et al., 2020). The secretory products of 3D-cultured cells are 10–100 times that of 2D-cultured cells, making it the ideal method for culturing exosomes at present (Watson et al., 2016). So far, 3D culture technology has been applied to the cultivation of hUCMSCs, BMSC, AMSCs, etc. However, the experience in culturing ASCs is still very limited (Lv et al., 2024; Wu et al., 2025; Ueyama et al., 2023). We established a 3D hollow fiber bioreactor system of ASCs (Figure 2A). We compared the difference in the number of exosomes harvested from 2D culture systems and 3D culture systems. The results indicated that when the same number of cells (approximately 1.2 × 10^7^) were cultured, the total amount of exosomes collected at one time in the 3D culture system was basically the same as that in the 2D culture system. However, culturing these cells in a 2D system required 30 culture flasks each time, with each flask needing 15–20 mL of culture medium; while in a 3D system, only about 20 mL was needed. Therefore, the volume of culture medium required for 3D culture systems was nearly 30 times less than that for 2D culture systems. The results showed that when the number of ASCs was the same, the total amount of exosomes collected once from the 3D culture system was basically the same as that from the 2D culture system, but the volume of the culture medium was nearly 30 times less (Figure 2B). In addition, In the 2D culture system, the maximum harvest frequency of exosomes was 3–4 times, while in the 3D culture system it was 30 times (Figure 2C). In our research, compared with the 2D culture system, the 3D culture system had advantages such as a large yield, less consumables, and convenient operation, which could support subsequent large-scale *in vivo* and *in vitro* experiments for the treatment of glioma. To identified the biological functions of ASC-Exos collected in a 3D environment. First, we respectively identified the supernatants harvested from the 2D culture system and the 3D culture system. The ELISA results of *Chlamydia* antibodies showed that the supernatants of both ASCs were negative (Attached Supplementary Table S1). The PCR results of *Mycoplasma* marker genes showed that the supernatants of both ASCs were negative (Figure 2D). The above results indicated that the supernatants harvested from both culture systems were free of *Chlamydia* and *Mycoplasma* contamination. Subsequently, we collected 2D-ASC-Exos and 3D-ASC-Exos by gradient ultracentrifugation and identified them. The results of transmission electron microscopy observation showed that the two types of ASC-Exos were in the form of hollow vesicles and had a double-layer lipid membrane structure (Figure 2E). The results of nanoparticle tracking analysis showed that the average particle diameters of the two ASC-Exos were both around 75 nm, which was within the common range of exosome particle sizes (30–150 nm) (Figure 2F). After standard calibration, the sample concentrations of 2D-ASC-Exos and 3D-ASC-Exos were 5 × 10^8^ and 1.77 × 10^10^ particles/mL, respectively. The protein contents of 2D-ASC-Exos and 3D-ASC-Exos determined by BCA were 2 × 10^3^ μg/mL and 6 × 10^4^ μg/mL, respectively. This was equivalent to approximately 4 × 10^−6^ μg and 3.39 × 10^−6^ μg of protein for each 2D-ASC-Exos and 3D-ASC-Exos, respectively. Nanoflow cytometry results showed that both types of ASC-Exos expressed the exosome marker proteins CD9 and CD63 (Figure 2G). The WB results indicated that both ASC-Exos expressed exosome marker proteins CD63, CD9, TSG101 and Calnexin (Figure 2H). The above results indicated that, compared with 2D-ASC-Exos, the morphology and properties of 3D-ASC-Exos were normal. These findings lay a solid foundation for exploring the therapeutic potential of 3D-ASC-Exos as drug delivery carriers in cancer treatment.

### 3.3 MiRNA expression profiling of 3D-ASC-Exos

**FIGURE 3:**
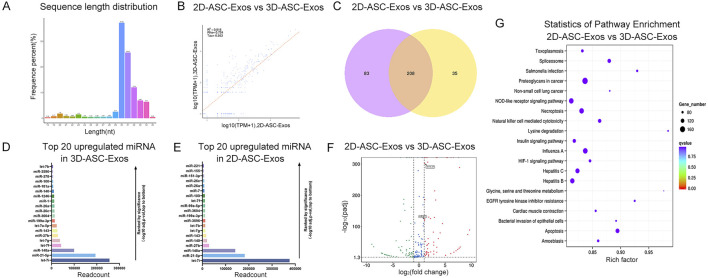
MiRNA expression profile analysis of 3D-ASC-Exos. **(A)** Length distribution of miRNAs in 3D-ASC-Exos. **(B)** Correlation analysis of 3D-ASC-Exos and 2D-ASC-Exos. **(C)** Venn diagram showing miRNAs of the two ASC-Exos. **(D)** TPM values of the top 20 highly expressed miRNAs in 3D-ASC-Exos. **(E)** TPM values of the top 20 highly expressed miRNAs in 2D-ASC-Exos. **(F)** Volcano plot showed differentially expressed miRNAs of the two ASC-Exos. **(G)** KEGG enrichment analysis of target genes of differentially expressed miRNAs. Data were presented as mean ± SD. One-way ANOVA combined with Tukey’s HSD test and Bonferroni correction was used. *P < 0.05, **P < 0.01, ***P < 0.001.

To evaluate the miRNAs expression profile of 3D-ASC-Exos, we performed Small RNA sequencing on them. And compared with our previous sequencing results of 2D-ASC-Exos. The results showed that the miRNAs of 3D-ASC-Exos were mainly distributed in the range of 30–32 nt in length, while those of 2D-ASC-Exos were mainly around 31 nt (Figure 3A). The sample correlation analysis results showed that the correlation coefficient was 0.516 (Figure 3B). The Venn diagram results showed that 208 miRNAs were common miRNAs of the two ASC-Exos (Figure 3C). Furthermore, among the top 20 highly expressed miRNAs of the two ASC-Exos, 15 miRNAs were consistent (Figures 3D,E). The results of differential miRNAs analysis showed that a total of 200 miRNAs were differentially expressed, among which 98 miRNAs were upregulated and 102 miRNAs were downregulated (Figure 3F). Among them, some upregulated miRNAs were closely related to the regulation of glioma cells. For instance, let-7a-5P has the function of inhibiting the proliferation of glioma and suppressing the stemness of glioma cells (Chen et al., 2022); miR-451 can inhibit the proliferation of glioma cells and also reduce the resistance of glioma cells to TMZ through the mTOR signaling pathway (Ogawa et al., 2019; Zhao et al., 2017). KEGG enrichment analysis was performed on the target genes of differentially expressed miRNAs (Figure 3G). Notably, the top 20 significantly different KEGG pathways were mostly related to tumor regulatory mechanisms such as uncontrolled proliferation, apoptosis resistance, immune escape, and metabolic reprogramming, including Proteoglycans in cancer, Non-small cell lung cancer, HIF-1 signaling pathway, Apoptosis, EGFR tyrosine kinase inhibitor resistance, Natural killer cell mediated cytotoxicity, Necroptosis, Spliceosome, NOD-like receptor signaling pathway, Glycine, serine and threonine metabolism (Kizhakkeppurath Kumaran et al., 2023; Mithoowani and Febbraro, 2022; Liu et al., 2021; Moyer et al., 2025; de Miguel et al., 2023; Bushnell et al., 2024; Zhou et al., 2023). Overall, compared with 2D-ASC-Exos, 3D-ASC-Exos were enriched with a higher proportion of miRNAs related to tumor regulation, suggesting that they have greater application potential in tumor treatment.

### 3.4 *In vitro* evaluation of the effects of 3D-ASC-Exos loaded with TMZ on the proliferation, migration, invasion and apoptosis of glioma cells

**FIGURE 4:**
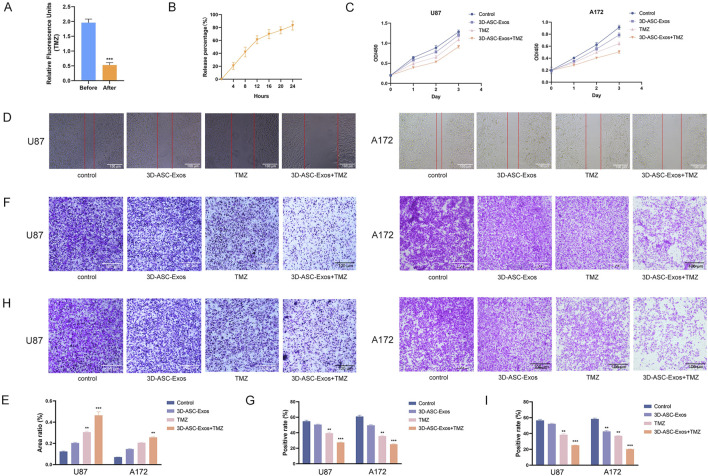
The effects of 3D-ASC-Exos loaded with TMZ on the proliferation, migration and invasion of glioma cells. **(A)** Relative fluorescence units of TMZ before and after ultracentrifugation (n = 3). **(B)** *In vitro* release profile of free TMZ in PBS for 24 h (n = 3). **(C)** CCK8 assay for the effect of 3D-ASC-Exos loaded with TMZ on the viability of U87 and A172 cells (n = 3). **(C)** CCK8 assay for the effect of 3D-ASC-Exos loaded with TMZ on the viability of U87 and A172 cells (n = 3). **(D)** Cell scratch for the effect of 3D-ASC-Exos loaded with TMZ on the migration ability of U87 and A172 cells. **(E)** Quantitative analysis of the scratch area (n = 3). **(F)** Transwell assay for the effect of 3D-ASC-Exos loaded with TMZ on the migration ability of U87 and A172 cells. **(G)** Quantitative analysis of the cell migration level (n = 3). **(H)** Transwell assay for the effect of 3D-ASC-Exos loaded with TMZ on the invasion ability of U87 and A172 cells. **(I)** Quantitative analysis of the cell invasion level (n = 3). Data were presented as mean ± SD. One-way ANOVA combined with Tukey’s HSD test and Bonferroni correction was used. *P < 0.05, **P < 0.01, ***P < 0.001.

**FIGURE 5:**
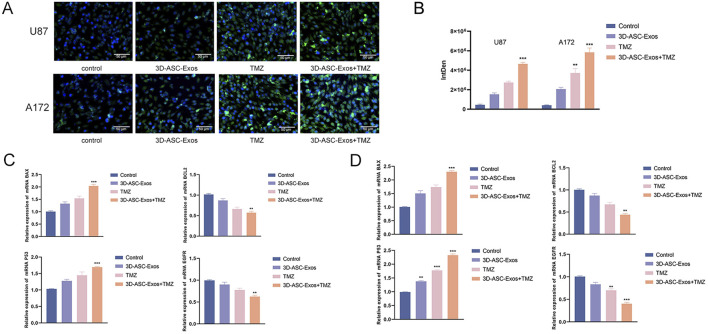
The effect of 3D-ASC-Exos loaded with TMZ on glioma cells. **(A)** TUNEL assay for the effect of 3D-ASC-Exos loaded with TMZ on the apoptosis ability of U87 and A172 cells. **(B)** Quantitative analysis of apoptosis levels (n = 3). **(C)** Q-PCR detection of the expression levels of BCL2, EGFR, P53, and BAX genes in U87 cells (n = 3). **(D)** Q-PCR detection of the expression levels of BCL2, EGFR, P53, and BAX genes in A172 cells (n = 3). The relative gene expression levels were calculated using the 2^-ΔΔCt^ method. Data were presented as mean ± SD. One-way ANOVA combined with Tukey’s HSD test and Bonferroni correction was used. *P < 0.05, **P < 0.01, ***P < 0.001.

The fluorescence intensity comparison indicated that approximately 76.9% of TMZ was encapsulated in 3D-ASC-Exos (Figure 4A). Moreover, the TMZ encapsulated in 3D-ASC-Exos could be gradually released into the external environment over time. By 24 h, approximately 83% of the TMZ had been released into the external environment (Figure 4B). We evaluated the effect of 3D-ASC-Exos loaded TMZ on inhibiting glioma *in vitro*. The CCK8 assay results indicated that the inhibitory effect of 3D-ASC-Exos loaded with TMZ on the proliferation ability of glioma cells was superior to that of using 3D-ASC-Exos or TMZ alone (Figure 4C). The cell scratch and Transwell results indicated that the inhibitory effect of 3D-ASC-Exos loaded with TMZ on the migration ability of glioma cells was superior to that of using 3D-ASC-Exos or TMZ alone, and the synergistic effect had more therapeutic advantages (Figures 4D–G). The Transwell assay results indicated that 3D-ASC-Exos loaded with TMZ significantly inhibited the invasion ability of glioma cells (Figures 4H,I). The TUNEL results showed that the 3D-ASC-Exos loaded with TMZ had the highest fluorescence intensity, indicating that it had the best effect in promoting glioma cell apoptosis (Figures 5A,B). The Q-PCR results indicated that 3D-ASC-Exos loaded with TMZ significantly inhibited the expression of the proto-oncogenes BCL2 and EGFR, and promoted the expression of the tumor suppressor genes P53 and BAX (Figures 5C,D). The above results indicated that 3D-ASC-Exos loaded with TMZ had the effects of inhibiting the proliferation, migration and invasion of glioma cells and promoting apoptosis. The application of exosomes as a nanocarrier system in tumor therapy had attracted extensive attention from scholars. Wei et al. found that compared with pure drug treatment, exosomes derived from MSCs carrying doxorubicin had a better inhibitory effect on the proliferation of osteosarcoma cells *in vitro* and were less toxic to the cells (Wei et al., 2019). Bagheri et al. found that exosomes derived from MSCs loaded with docetaxel could highly accumulate at tumor sites and significantly inhibit tumor growth. Meanwhile, compared with the sole use of docetaxel, the docetaxel-loaded exosomes derived from MSCs were metabolized by the liver at a faster rate (Bagheri et al., 2020). Despite a large number of studies confirmed that MSC-Exos, as drug carriers, possess numerous advantages in cancer treatment, they could efficiently inhibit tumor cell proliferation, promote tumor cell apoptosis, and enhance drug efficacy. However, in the complex physiological and pathological environment, the functions of MSC-Exos were not constant. In recent years, there have been reports that exosomes derived from MSCs can also exhibit promoting effects on cancer cells. Zhao et al. discovered that exosomes derived from BMSCs promoted the proliferation and migration of and osteosarcoma cells, as well as tumor occurrence (Zhao et al., 2019). Ren et al. found that BMSC-Exos pretreated with hypoxia inhibited the expression levels of pro-apoptotic genes (such as PTEN, PDCD4 and RECK) through miR-21-5p, thereby slowing down the apoptosis process of lung cancer cells (Ren et al., 2019). Therefore, it is necessary for us to find more exosomes derived from mesenchymal stem cells for tumor treatment. ASCs were the basis for the rapid growth of antler without causing cancer. As a novel type of MSCs, ASCs had demonstrated significant advantages in targeted glioma therapy due to their inherent anti-glioma properties and load-bearing plasticity.

### 3.5 *In vivo* evaluation of the safety and efficacy of 3D-ASC-Exos loaded with TMZ in anti-glioma therapy

**FIGURE 6:**
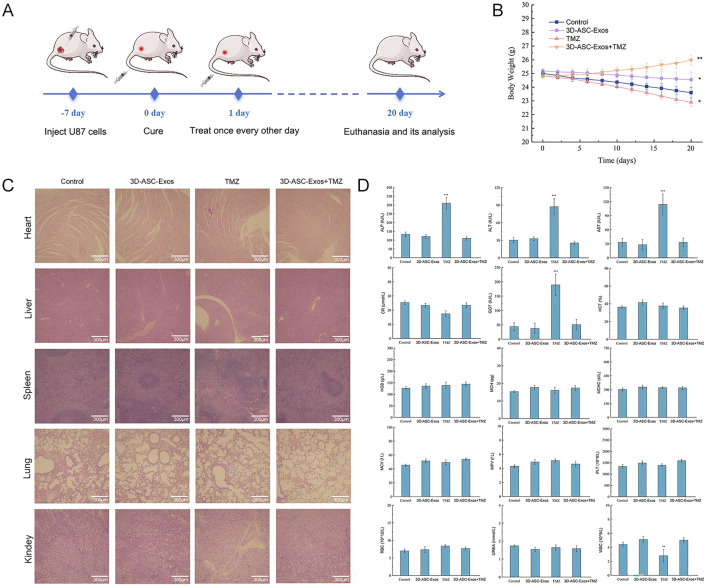
Safety evaluation of 3D-ASC-Exos loaded with TMZ in anti-glioma treatment. **(A)** Schematic diagram of the experimental design showed the establishment of a glioma nude mouse model and subsequent 3D-ASC-Exos treatment. **(B)** Trend chart of nude mouse body weight changes (n = 3). **(C)** HE staining of nude mouse internal organs. **(D)** Routine blood and blood biochemical analysis of nude mice (n = 3). Tumor volume = length × width^2^/2. Data were presented as mean ± SD. One-way ANOVA combined with Tukey’s HSD test and Bonferroni correction was used. *P < 0.05, **P < 0.01, ***P < 0.001.

**FIGURE 7:**
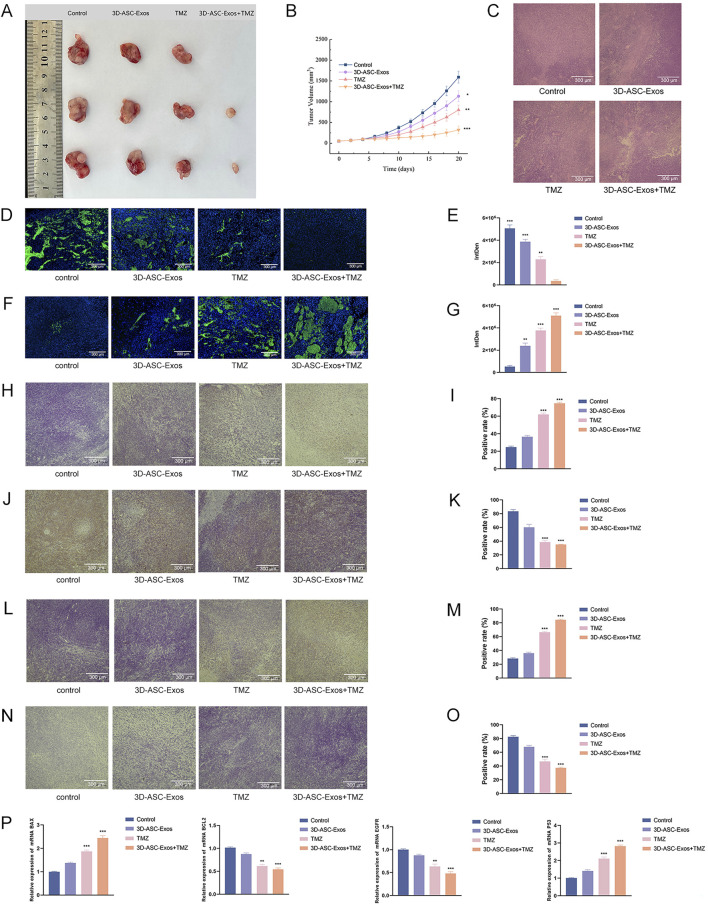
Evaluation of the anti-glioma efficacy of 3D-ASC-Exos loaded with TMZ. **(A)** Gross observation of tumors. **(B)** Trend of tumor volume changes. **(C)** HE staining of tumor tissues (n = 3). **(D)** Ki67 fluorescence staining of tumors. **(E)** Quantitative analysis of Ki67 staining (n = 3). **(F)** TUNEL staining of tumors. **(G)** Quantitative analysis of TUNEL staining (n = 3). **(H)** Immunohistochemical detection of BAX expression in tumor tissues. **(I)** Quantitative analysis of BAX (n = 3). **(J)** Immunohistochemical detection of BCL2 expression in tumor tissues. **(K)** Quantitative analysis of BCL2 (n = 3). **(L)** Immunohistochemical detection of P53 expression in tumor tissues. **(M)** Quantitative analysis of P53 (n = 3). **(N)** Immunohistochemical detection of EGFR expression in tumor tissues. **(O)** Quantitative analysis of EGFR (n = 3). **(P)** Q-PCR analysis of the expression levels of BAX, BCL2, P53, and EGFR (n = 3). The relative gene expression levels were calculated using the 2^-ΔΔCt^ method. Data were presented as mean ± SD. One-way ANOVA combined with Tukey’s HSD test and Bonferroni correction was used. *P < 0.05, **P < 0.01, ***P < 0.001.

An increasing amount of clinical evidence indicated that exosomes could not only enhance the therapeutic effect of drugs but also reduce the systemic toxicity of chemotherapy drugs (Liang et al., 2021). To evaluate the safety of 3D-ASC-Exos loaded TMZ *in vivo* against glioma, 3D-ASC-Exos were used to treat U87 glioma nude mice (Figure 6A). On the seventh day after subcutaneous injection of U87 cells in nude mice, PBS, 3D-ASC-Exos (500 μg/mL), TMZ (50 μg/mL), and 3D-ASC-Exos + TMZ (500 μg/mL) were respectively administered via tail vein injection of 0.1 mL every other day. During the 20-day treatment period, the body weight of nude mice in the 3D-ASC-Exos + TMZ group not only did not decrease but also showed a slight upward trend (Figure 6B). The results of HE staining of the internal organs of mice showed that 3D-ASC-Exos loaded with TMZ had no damage to the heart, liver, spleen, lung and kidney of mice after treatment (Figure 6C). Routine blood tests and blood biochemical analyses revealed that the levels of ALP, ALT, AST, and GGT in the TMZ group significantly increased, while the WBC count decreased. However, the values of each group in the 3D-ASC-Exos + TMZ group and the 3D-ASC-Exos group showed no difference from those in the Control group (Figure 6D). The results indicated that the treatment method of 3D-ASC-Exos loaded with TMZ had no effect on the physiological indicators and liver-kidney functions of nude mice with glioma. The above results suggested that 3D-ASC-Exos had good systemic safety and biocompatibility. In addition, 3D-ASC-Exos loaded with TMZ in the treatment of glioma-bearing nude mice reduced the damage of chemotherapy drugs to the body. We further evaluated the *in vivo* anti-glioma effect of 3D-ASC-Exos loaded with TMZ. Overall observation results indicated that the tumors in the 3D-ASC-Exos + TMZ group were significantly smaller than those in the TMZ group (Figures 7A,B). Even the tumor of one of them had disappeared, indicating that the tumor of this nude mouse had been cured. The surfaces of the grayish-white tumors of the other two mice also showed almost no blood vessels, indicating that the tumor development process of these two nude mice was significantly inhibited. The HE staining results of the tumor tissues showed that in the 3D-ASC-Exos + TMZ group, the surface fissures of the slices spread from the center to every part of the tumor, and dense fissures and vacuoles appeared inside the tumor. Even the deep-stained areas were no longer full (Figure 7C). The above results indicated that the tumor had begun to disintegrate internally. The Ki67 fluorescence staining results of tumor sections showed that in the 3D-ASC-Exos + TMZ group, the positive staining area almost disappeared, with only a few scattered positive staining spots remaining (Figures 7D,E). The result indicated that the tumor’s proliferative capacity had almost vanished at this time. The total fluorescence intensity of the positive area in each group was used as the quantitative value. The TUNEL staining results indicated that in the 3D-ASC-Exos + TMZ group, the positive areas had occupied the entire section, and the positive cells were connected together, suggesting that severe apoptosis had occurred in the tumor tissue at this time, and the degree of apoptosis was more severe compared to the TMZ group (Figures 7F,G). The immunohistochemistry and Q-PCR results indicated that 3D-ASC-Exos loaded with TMZ significantly downregulated the expression of the oncogenes BCL2 and EGFR, and promoted the expression of the tumor suppressor genes P53 and BAX (Figures 7H–P). The above results suggested that 3D-ASC-Exos loaded with TMZ could effectively inhibit the development process of glioma. In conclusion, 3D-ASC-Exos had shown excellent anti-cancer effects as drug delivery carriers and had mitigated the adverse effects on the body caused by the intake of pure drugs. It was worth noting that in this study, since the tumor-bearing mice were subjected to ectopic modeling, there might be differences compared to the actual therapeutic effects of glioma.

## 4 Conclusion

This study established a 3D hollow fiber bioreactor system for ASCs culture. The total amount and concentration of exosomes obtained by this method were 10 times and 30 times those obtained by the 2D method, respectively. 3D-ASC-Exos had the same morphology and properties as 2D-ASC-Exos. However, 3D-ASC-Exos were enriched with more miRNAs related to tumor suppression. Due to the subcutaneous inoculation modeling method lacking a complete blood-brain barrier structure, drugs could directly contact tumor cells. Therefore, this manuscript was more suitable for evaluating the direct anti-tumor effect of drugs, and at the same time, it could more truly reflect the systemic toxicity and metabolic characteristics of the drugs. In future research, we will conduct more in-depth studies on whether 3D-ASC-Exos carrying TMZ can penetrate the blood-brain barrier. Both *in vitro* and *in vivo* studies had shown that 3D-ASC-Exos loaded with TMZ could inhibit the proliferation, migration and invasion of glioma cells and promote apoptosis, thereby exerting anti-glioma effects. Meanwhile, 3D-ASC-Exos had excellent systemic safety and biocompatibility, which could reduce the damage of chemotherapy drugs to the body. The development of 3D-ASC-Exos as a drug delivery carrier not only offers a superior tumor treatment strategy but also demonstrates the broad potential of exosomes in targeted tumor therapy.

## Data Availability

The datasets presented in this study can be found in online repositories. The names of the repository/repositories and accession number(s) can be found in the article/Supplementary Material.
