# Supplementary material for: 3D-ASC-Exos as a novel drug carrier for glioma treatment
Source: Front Cell Dev Biol. 2025 Sep 25;13:1677555. doi: 10.3389/fcell.2025.1677555 (PMC12507771; doi:10.3389/fcell.2025.1677555)
Supplement: Supplementary file 1 [file Table1.docx]

**Supplementary Information**

**3D-ASC-Exos as a novel drug carrier for glioma treatment**

a College of Agriculture, Yanbian University, Yanji, China

b College of Animal Science and Technology, Jilin Agricultural University, Changchun City, China

c Jilin Province Sika Deer Efficient Breeding and Product Development Technology Engineering Research Center, Jilin Agricultural University, Changchun, China.

**Experimental section**

**Table S1**

Chlamydia in the supernatant of 2D-ASCs

|  | **Average value of negative control** | **Average value of positive control** | **Supernatant of ASCs** |
| --- | --- | --- | --- |
| OD450 | 0.06 | 3.56 | 0.06 |

Chlamydia trachomatis in 3D-ASCs

|  | **Average value of negative control** | **Average value of positive control** | **Supernatant of ASCs** |
| --- | --- | --- | --- |
| OD450 | 0.09 | 2.94 | 0.22 |
